# Evaluation of the Antifibrotic Effects of Drugs Commonly Used in Inflammatory Intestinal Diseases on In Vitro Intestinal Cellular Models

**DOI:** 10.3390/ijms25168862

**Published:** 2024-08-14

**Authors:** Serena Artone, Alessia Ciafarone, Francesca Rosaria Augello, Francesca Lombardi, Maria Grazia Cifone, Paola Palumbo, Benedetta Cinque, Giovanni Latella

**Affiliations:** 1 Department of Life, Health & Environmental Sciences, University of L’Aquila, Via Pompeo Spennati, Building Rita Levi Montalcini, Coppito, 67100 L’Aquila, Italy; serena.artone@graduate.univaq.it (S.A.); alessia.ciafarone@graduate.univaq.it (A.C.); francescarosaria.augello@univaq.it (F.R.A.); francesca.lombardi@univaq.it (F.L.); mariagrazia.cifone@univaq.it (M.G.C.); paola.palumbo@univaq.it (P.P.); benedetta.cinque@univaq.it (B.C.); 2 PhD School in Medicine and Public Health, Department of Life, Health and Environmental Sciences, University of L’Aquila, 67100 L’Aquila, Italy; 3 PhD School in Health & Environmental Sciences, Department of Life, Health and Environmental Sciences, University of L’Aquila, 67100 L’Aquila, Italy; 4 Unit of Gastroenterology, Hepatology, and Nutrition, Department of Life, Health & Environmental Sciences, University of L’Aquila, Via Pompeo Spennati, Building Rita Levi Montalcini, Coppito, 67100 L’Aquila, Italy

**Keywords:** CCD-18Co cells, Caco-2 IEC, TGF-β1, Smad 2/3, collagen-I, intestinal fibrosis, α-SMA, occludin, EMT, IBD

## Abstract

The mechanism underlying intestinal fibrosis, the main complication of inflammatory bowel disease (IBD), is not yet fully understood, and there is no therapy to prevent or reverse fibrosis. We evaluated, in in vitro cellular models, the ability of different classes of drugs currently used in IBD to counteract two pivotal processes of intestinal fibrosis, the differentiation of intestinal fibroblasts to activated myofibroblasts using CCD-18Co cells, and the epithelial-to-mesenchymal transition (EMT) of intestinal epithelial cells using Caco-2 cells (IEC), both being processes induced by transforming growth factor-β1 (TGF-β1). The drugs tested included mesalamine, azathioprine, methotrexate, prednisone, methylprednisolone, budesonide, infliximab, and adalimumab. The expression of fibrosis and EMT markers (collagen-I, α-SMA, pSmad2/3, occludin) was assessed by Western blot analysis and by immunofluorescence. Of the drugs used, only prednisone, methylprednisolone, budesonide, and adalimumab were able to antagonize the pro-fibrotic effects induced by TGF-β1 on CCD-18Co cells, reducing the fibrosis marker expression. Methylprednisolone, budesonide, and adalimumab were also able to significantly counteract the TGF-β1-induced EMT process on Caco-2 IEC by increasing occludin and decreasing α-SMA expression. This is the first study that evaluates, using in vitro cellular models, the direct antifibrotic effects of drugs currently used in IBD, highlighting which drugs have potential antifibrotic effects.

## 1. Introduction

Intestinal fibrosis is a common complication of inflammatory bowel disease (IBD), including Crohn’s Disease (CD), which may affect the entire gastrointestinal tract, and Ulcerative Colitis (UC), limited to the large bowel [1]. Several factors, including environment, gut microbiome, genetics, and immune disorders, are involved in the onset, maintenance, and progression of IBD [2]. Fibrosis always develops at the level of the inflamed intestinal segments, suggesting an inflammation–fibrosis sequence process with permanent damage up to stenosis and obstruction [3]. Intestinal fibrosis is characterized by excessive extracellular matrix (ECM) deposition, including fibronectin and collagens. It depends on the imbalance between the production and degradation of ECM proteins induced by a complex and dynamic interaction between profibrotic and antifibrotic mediators [4]. ECM is produced by activated myofibroblasts, whose progenitors include resident mesenchymal cells (fibroblasts, sub-epithelial myofibroblasts, and smooth muscle cells), pericytes, and stellate cells. Additionally, a further source of myofibroblasts is the endothelial–mesenchymal transition (EndMT) and epithelial–mesenchymal transition (EMT), processes through which endothelial and epithelial cells lose their features and functions and acquire a mesenchymal cell phenotype [5,6]. In particular, the EMT process leads to a gradual loss of epithelial markers (E-cadherin, occludin) followed by the upregulation in the expression of mesenchymal markers (α-SMA, vimentin) [7]. Several studies indicated that EMT is involved in the intestinal fibrosis process [8,9]. Experimental studies with transgenic mouse models showed that about one-third of fibroblast-specific protein 1 (FSP1)^+^ fibroblasts derived from intestinal epithelial cells [8]. Furthermore, Masià et al. [10] showed that EMT is increased in intestinal specimens from CD patients with a penetrating behavior. Transforming growth factor-β1 (TGF-β1) is the main regulator of intestinal fibrosis, and it is overexpressed both in the intestinal fibrosis of CD patients and animal models of IBD. TGF-β1 signaling is mediated by canonical (Smads) and non-canonical (MAP kinase pathways, Rho-like GTPase signaling pathways, and phosphatidylinositol-3-kinase/AKT pathway) pathways [4]. The canonical TGF-β1 signaling pathway uses Smad2 and/or Smad3 to transfer signals. TGF-βR1 directly phosphorylates Smad2/3, binds to Smad4, and translocates into the nucleus to regulate the gene transcription involved in fibrosis [11]. The development of intestinal fibrosis is a complex process, whose specific molecular mechanisms have not yet been fully defined. Although chronic inflammation is the trigger factor of fibrosis, its progression becomes an independent process. In fact, the control of inflammation is not enough to reverse the process. All anti-inflammatory drugs, even the most potent biological drugs, have limited and transient antifibrotic effects. To date, effective drugs in preventing and treating fibrosis are not yet available. 

We evaluated, using in vitro cellular models, the ability of different classes of drugs currently used in IBD to counteract two pivotal processes of intestinal fibrosis, including the differentiation of intestinal fibroblasts to activated myofibroblasts using the CCD-18Co cell line, and the epithelial-to-mesenchymal transition (EMT) of intestinal epithelial cells using the Caco-2 cell line (IEC), with both processes induced by TGF-β1. The drugs tested included the mesalamine (5-aminosalicylic acid (5-ASA)), azathioprine, prednisone, methylprednisolone, budesonide, methotrexate, and anti-TNFα antibodies (infliximab and adalimumab).

## 2. Results

### 2.1. Effect of Selected Drugs on Cell Viability of CCD-18Co and IEC Caco-2

**Figure 1:**
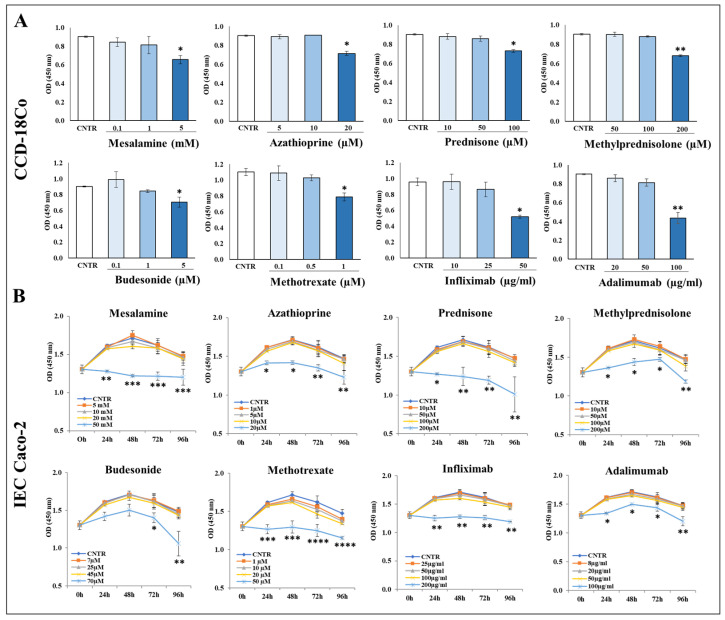
Cell viability of CCD-18Co and IEC Caco-2. (**A**) Cell viability of CCD-18Co cells incubated for 48 h with mesalamine (0.1–5 mM), azathioprine (5–20 µM), prednisone (10–100 µM), methylprednisolone (50–200 µM), budesonide (0.1–5 µM), methotrexate (0.1–1 µM), infliximab (10–50 µg/mL), and adalimumab (20–100 µg/mL). (**B**) IEC Caco-2 cells incubated up to 96 h with mesalamine (5–50 mM), azathioprine (1–20 µM), prednisone (10–200 µM), methylprednisolone (10–200 µM), budesonide (7–70 µM), methotrexate (1–50 µM), infliximab (25–200 µg/mL), and adalimumab (8–100 µg/mL). The data are from two independent experiments performed in triplicate, and values are expressed as mean ± SEM. For comparative analysis of data, a one-way ANOVA for CCD-18Co cells and two-way ANOVA for IEC Caco-2 cells followed by Dunnett’s post hoc test were performed. * *p* < 0.05, ** *p* < 0.01, *** *p* < 0.001, **** *p* < 0.0001.

The effect of the selected drugs on the cell viability of CCD-18Co and IEC Caco-2 cells was evaluated by a Cell Counting Kit-8 (CCK-8). As shown, in CCD-18Co cells, all drugs at 48 h significantly reduced the absorbance associated with a cell number decrease at the highest dose tested compared to controls (Figure 1A). Similarly, in IEC Caco-2 cells, cell number was significantly reduced only at the highest dose of the tested drugs (Figure 1B). Based on these results, the highest dose of each drug was excluded from the subsequent experiments. 

Since the tumor necrosis factor-α (TNF-α) is the target of some selected drugs, its levels in the cell culture supernatant were assessed by a specific ELISA kit assay. TNF-α levels in Caco-2 IEC cells were 30 ± 7.4 pg/10^5^ cells, while in CCD-18Co, levels were 290 ± 4.0 pg/10^5^ cells. TNF-α concentration is expressed as mean ± SEM of two independent experiments performed in duplicate and normalized per 10^5^ cells.

### 2.2. Effect of Selected Drugs on TGB-β1-Induced Collagen I and α-SMA Expression in CCD-18Co Cells

**Figure 2:**
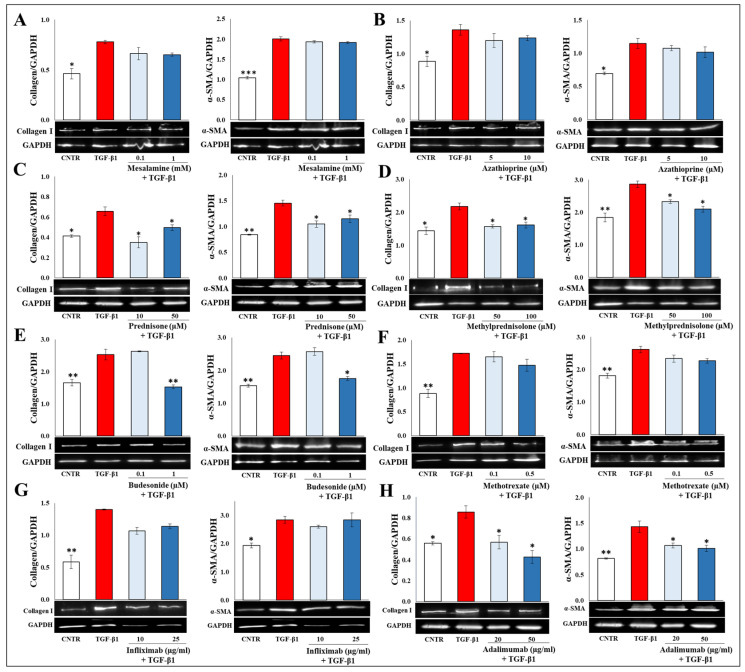
Effect of tested drugs on TGB-β1-induced collagen I and α-SMA expression in CCD-18Co cells. Immunoblotting assay for collagen I and α-SMA was performed on CCD-18Co cells incubated for 48 h with TGF-β1 (10 ng/mL) in the presence or absence of (**A**) mesalamine (0.1–1 mM), (**B**) azathioprine (5–10 µM), (**C**) prednisone (10–50 µM), (**D**) methylprednisolone (50–100 µM), (**E**) budesonide (0.1–1 µM), (**F**) methotrexate (0.1–0.5 µM), (**G**) infliximab (10–25 µg/mL), and (**H**) adalimumab (20–50 µg/mL). Representative images of immunoblotting for collagen I (130 kDa), α-SMA (42 kDa), and GAPDH (37 kDa) are shown. The data are from three independent experiments, and values are expressed as mean ± SEM. For comparative analysis of data, a one-way analysis of variance (ANOVA) with Dunnett’s post hoc test was used. Densitometric analysis was performed by normalizing vs. GAPDH. * *p* < 0.05, ** *p* < 0.01, *** *p* < 0.001.

Differentiation of fibroblasts to myofibroblasts is an essential cellular event in intestinal fibrosis. It is mainly characterized by a relevant cytoskeleton organization and an increased expression of fibrotic markers, such as collagen-I and the α-SMA protein. Western blot analysis was performed to evaluate the effects of selected drugs on collagen I and α-SMA expression levels. As shown in Figure 2, the CCD-18Co cells, serum-deprived for 24 h and then stimulated with TGF-β1 (10 ng/mL) for 48 h, underwent a phenotypic transformation into activated myofibroblasts, characterized by a significant increase of collagen I and α-SMA expression compared to the untreated control cells. Among the tested drugs, mesalamine, azathioprine, methotrexate, and infliximab did not counteract the TGF-β1-induced effects in CCD-18Co cells. On the other hand, the steroids prednisone, methylprednisolone, budesonide, and adalimumab were able to moderately counteract the increase of collagen and α-SMA expression induced by TGF-β1. 

**Figure 3:**
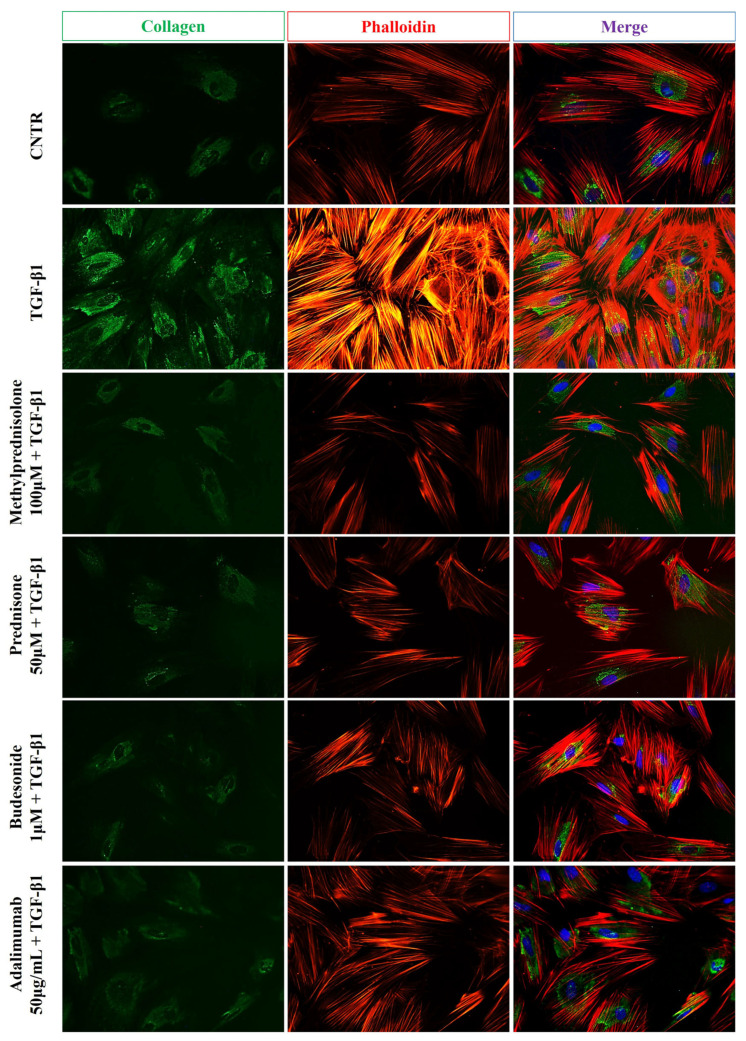
Effect of drugs on TGB-β1-induced collagen I expression in CCD-18Co cells. Representative immunofluorescence images of untreated and treated CCD-18Co cells, as above described, stained with anti-collagen I antibody (green) and TRITC-phalloidin (red) to reveal F-actin. Nuclei were counterstained with DAPI (blue) (magnification 40×). The images are representative of three independent experiments in duplicate.

Immunofluorescence staining was performed to further confirm the beneficial effect given by methylprednisolone, prednisone, budesonide, and adalimumab in CCD-18Co cells. As expected, the TGF-β1 treatment led to increased collagen I expression, shown by more intense staining in the treated cells compared to the untreated control cells. On the other hand, cells treated with TGF-β1 plus prednisone, methylprednisolone, budesonide, and adalimumab showed less intense collagen I staining, similar to that observed in untreated cells. Furthermore, TGF-β1-induced fibrotic activation is characterized by notable cytoskeletal rearrangement due to the development of actin stress fibers, as highlighted by intense phalloidin staining. Of note, the presence of the selected drugs dampened the intense and marked fluorescence of phalloidin, bringing it back to control levels (Figure 3).

**Figure 4:**
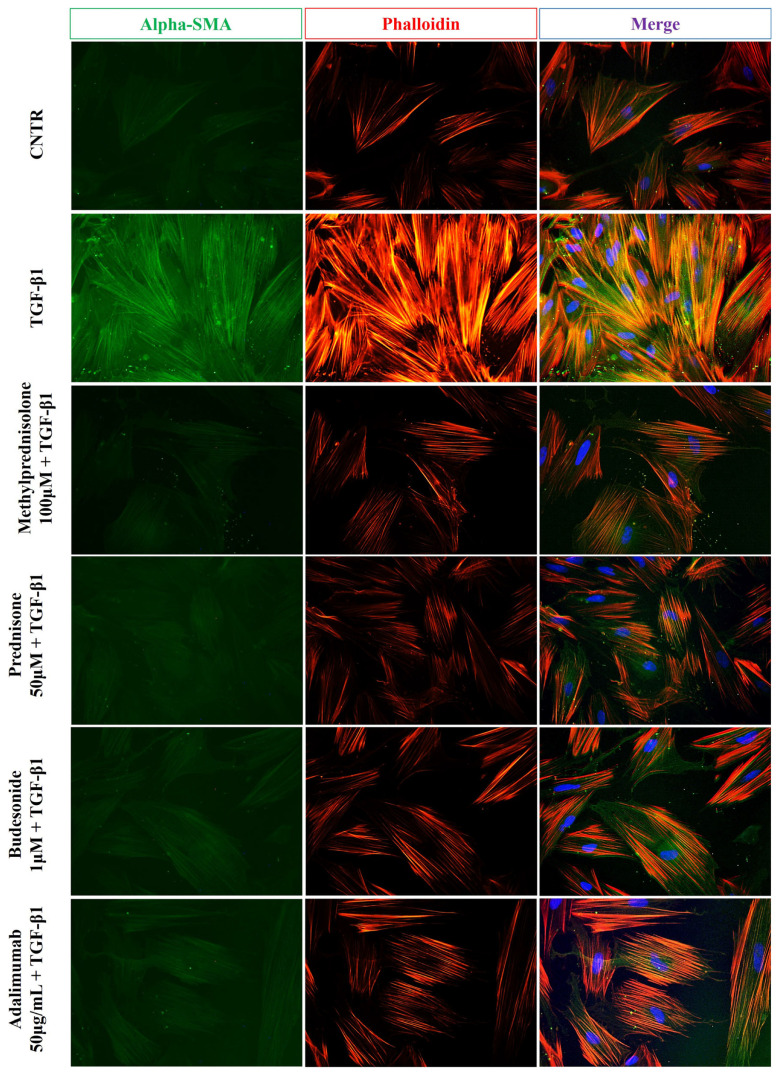
Effect of drugs on TGB-β1-induced α-SMA expression in CCD-18Co cells. Representative immunofluorescence images of untreated and treated CCD-18Co cells, as described above, stained with anti-α-SMA antibody (green) and TRITC-phalloidin (red) to reveal F-actin. Nuclei were counterstained with DAPI (blue) (magnification 40×). The images are representative of three independent experiments performed in duplicate.

Furthermore, as reported in Figure 4, immunofluorescence analysis showed that the green fluorescent signal relative to α-SMA expression was weak in untreated control cells and intense in activated myofibroblasts, confirming an increase of α-SMA protein level following TGF-β1 treatment. Once again, the treatment with methylprednisolone, prednisone, budesonide, and adalimumab was able to reverse the phenotype induced by TGF-β1 as shown by lower and weak fluorescence of α-SMA. 

### 2.3. Effect of Selected Drugs on TGB-β1-Induced Smad Signaling

**Figure 5:**
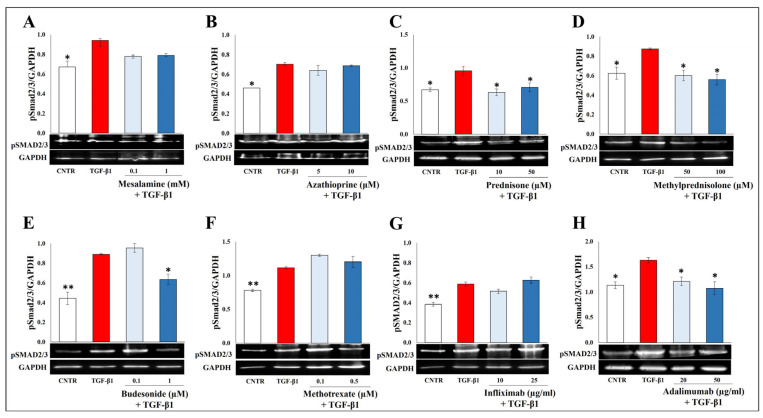
Effect of tested drugs on TGB-β1-induced Smad signaling in CCD-18Co cells. Immunoblotting assay for pSmad 2/3 was performed on CCD-18Co cells incubated for 48 h with TGF-β1 (10 ng/mL) in the presence or absence of (**A**) mesalamine (0.1–1 mM), (**B**) azathioprine (5–10 µM), (**C**) prednisone (10–50 µM), (**D**) methylprednisolone (50–100 µM), (**E**) budesonide (0.1–1 µM), (**F**) methotrexate (0.1–0.5 µM), (**G**) infliximab (10–25 µg/mL), and (**H**) adalimumab (20–50 µg/mL). Representative images of immunoblotting pSmad2/3 (52 kDa) and GAPDH (37 kDa) are shown. The data are from three independent experiments, and values are expressed as mean ± SEM. For comparative analysis of data, a one-way analysis of variance (ANOVA) with Dunnett’s post hoc test was used. Densitometric analysis was performed by normalizing vs. GAPDH. * *p* < 0.05, ** *p* < 0.01.

TGF-β1 activates the Smad2/3 pathway to exert its pro-fibrotic effects. As shown in Figure 5, the expression of p-Smad2/3 protein was significantly increased after TGF-β1 stimulation, and only prednisone, methylprednisolone, budesonide, and adalimumab were able to counteract this effect. On the other hand, the treatment with mesalamine, azathioprine, methotrexate, and infliximab failed to modify the phosphorylation of Smad2/3.

### 2.4. Effect of Selected Drugs on TGB-β1-Induced Epithelial to Mesenchymal Transition in Caco-2 IEC Cells

**Figure 6:**
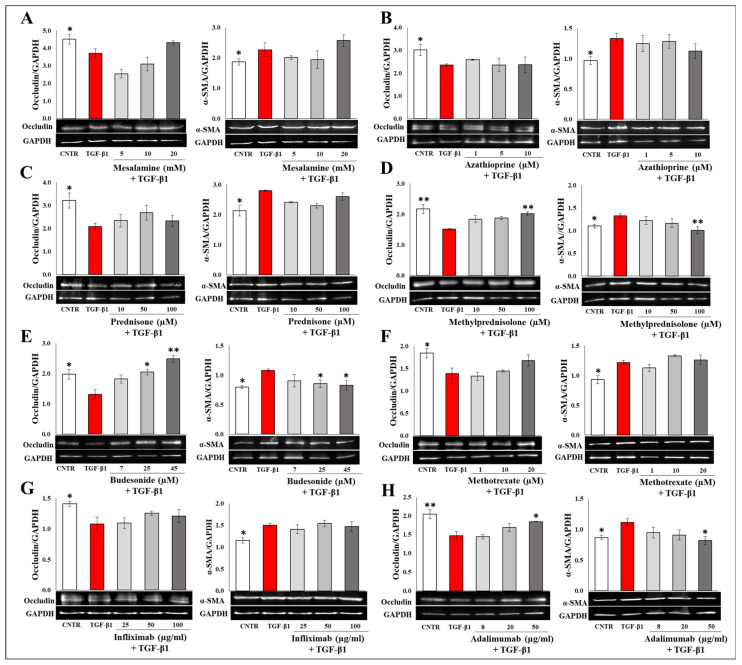
Effect of tested drugs on EMT in Caco-2 IEC cells. Immunoblotting assay for occludin and α-SMA was performed on Caco-2 IEC cells incubated for 96 h with TGF-β1 (20 ng/mL) in the presence or absence of (**A**) mesalamine (5–20 mM), (**B**) azathioprine (1–10 µM), (**C**) prednisone (10–100 µM), (**D**) methylprednisolone (10–100 µM), (**E**) budesonide (7–45 µM), (**F**) methotrexate (1–20 µM), (**G**) infliximab (25–100 µg/mL), and (**H**) adalimumab (8–50 µg/mL). Representative images of immunoblotting for occludin (59 kDa), α-SMA (42 kDa), and GAPDH (37 kDa) are shown. The data are from three independent experiments, and values are expressed as mean ± SEM. For comparative analysis of data, a one-way analysis of variance (ANOVA) with Dunnett’s post hoc test was used. Densitometric analysis was performed by normalizing vs. GAPDH. * *p* < 0.05, ** *p* < 0.01.

The EMT process, an important feature of fibrogenesis, is characterized by a decrease in epithelial marker expression and a subsequent increase of mesenchymal markers in intestinal epithelial cells. In particular, during EMT, a marked actin rearrangement occurs when cell–cell junctions are dismantled. Thus, to evaluate the in vitro effect of mesalamine, azathioprine, prednisone, methylprednisolone, budesonide, methotrexate, infliximab, and adalimumab on EMT, IEC Caco-2 cells were cultured for four days in the presence of 20 ng/mL TGF-β1 with or without the selected drugs. The expression levels of epithelial marker occludin, a transmembrane protein of the tight junction, and mesenchymal marker α-SMA were analyzed by Western blot. As expected, a decrease in the epithelial marker and an increase in the mesenchymal marker were observed in the presence of TGF-β1. Of the tested drugs, only methylprednisolone, budesonide, and adalimumab were able to significantly counteract the effect induced by TGF-β1 (Figure 6).

**Figure 7:**
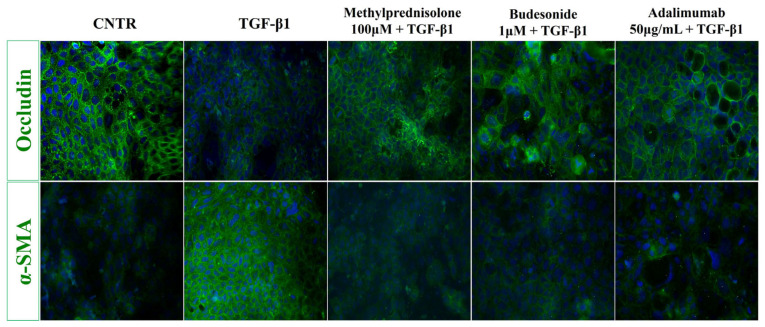
Effect of drugs on EMT markers (occludin and α-SMA expression) in Caco-2 IEC cells. Representative immunofluorescence images of untreated and treated Caco-2 IEC cells, as described above, stained with anti-occludin and anti-α-SMA antibodies (green). Nuclei were counterstained with DAPI (blue) (magnification 40×). The images are representative of three independent experiments in duplicate.

Subsequently, to further confirm the efficacy of methylprednisolone, budesonide, and adalimumab on EMT markers, immunofluorescence analysis was performed. As shown in Figure 7, the treatment of Caco-2 IEC cells with TGF-β1 resulted in a decreased level of occludin, as demonstrated by the slight staining, and an increased α-SMA expression, as observed by an intense green fluorescence. Of note, the treatment with the above drugs was able to counteract the TGF-β1-induced effect, with the levels of occludin and α-SMA comparable to the untreated cells.

## 3. Discussion

IBD is characterized by chronic and relapsing inflammation, with frequent development of fibrosis at the sites of inflammation and possible intestinal stenosis and obstruction. Intestinal fibrosis develops in approximately 30–50% of CD patients and 1–12% of UC patients [12]. The development of intestinal fibrosis is a multifactorial, long, complex, and progressive process characterized by an excessive and uncontrolled accumulation of ECM components, which causes tissue stiffness and leads to the expansion of smooth muscles, ending in intestinal obstruction [13,14]. Multiple cellular compartments drive intestinal fibrosis, including mesenchymal cells, fibroblasts, myofibroblasts, and smooth muscle cells. Myofibroblasts, the fibrotic process’s main actors, can also arise from non-mesenchymal cells, including epithelial and endothelial cells via EMT and EndMT [8]. Increased evidence has shown EMT to be the most important process of intestinal fibrosis, demonstrated by EMT-related molecules found in CD fibrotic lesions and fistula [15,16]. Activated mesenchymal cells, in addition to producing matrix components, also secrete chemokines, cytokines, and growth factors that recruit immune cells, thus fueling chronic inflammation [17]. In this regard, TGF-β1 plays a pivotal role in intestinal fibrosis development, stimulating the EMT process and driving the fibroblast differentiation into myofibroblasts to produce α-SMA, involved in their contractility, and ECM proteins such as collagen and fibronectin [18]. 

Although considerable progress has been made in controlling inflammation in IBD, intestinal fibrosis and stricture formation incidence remain high, probably because fibrosis, once initiated, is self-maintaining and progresses with mechanisms independent of inflammation. This has important implications in the treatment of IBD. It could explain why the currently used drugs, with their anti-inflammatory action, have poor effects both in preventing fibrosis and its reversal once it has occurred [18]. Different classes of drugs are currently used in the therapy of IBD, including salicylates (5-ASA), corticosteroids (prednisone, methylprednisolone, and budesonide), immunosuppressants (azathioprine, methotrexate, and cyclosporine), and biological drugs (infliximab, adalimumab, golimumab, certolizumab pegol, vedolizumab, and ustekinumab), all with specific anti-inflammatory actions, but their potential antifibrotic effects are poorly known. To date, specific antifibrotic therapies are not yet available.

In the present study, we evaluated, using in vitro cellular models, the ability of these different classes of drugs used in IBD to counteract two pivotal processes of intestinal fibrosis, including the differentiation of fibroblasts to activated myofibroblasts and the EMT induced by TGF-β1. TGF-β1 induced the differentiation of intestinal CCD-18Co fibroblasts into myofibroblasts and the EMT process in Caco-2 IEC cells. These effects were highlighted by the Western blot and immunofluorescence analyses, showing, on the one hand, in fibroblasts a significant increase of collagen I and α-SMA (fibrosis markers) expression, and on the other, in Caco-2 IEC cells a substantial decrease of occludin expression and a noticeable increase of α-SMA level (markers of the EMT process). Of note, the increased expression of elevated levels of α-SMA in activated myofibroblasts is functionally involved in their increased ability to contract and interact with the ECM during the fibrogenesis process [19]. The contractile ability of activated fibroblasts is produced through stress fibers, made of actin microfilament (F-actin) bundles, selectively labelled by phalloidin, able to bind with high affinity to the polymerized form of actin and not actin monomers (G-actin) [20]. When α-SMA is integrated into stress fibers, the contractility function is enhanced, generating distinct contacts with the ECM [21,22]. In the present study, the reorganization of the actin cytoskeleton in TGF-β1-treated CCD18-Co cells was demonstrated by more intense fluorescent staining of phalloidin than untreated control cells. Likewise, a more marked green staining of collagen I and α-SMA in the cells treated with TGF-β1 indicated the differentiation of fibroblasts to activated myofibroblasts. Consistent with these results, TGF-β1-treated CCD18-Co cells showed a significant increase in expression of the activated pSmad2/3. Furthermore, immunofluorescence analysis showed a slight and weak staining of occludin and a stronger fluorescence intensity of α-SMA in TGF-β1-treated Caco-2 IEC cells compared to untreated cells, indicating the occurrence of the EMT process. 

Of the conventional drugs, 5-ASA has been used for more than 50 years and has shown efficacy and safety in inducing and maintaining clinical and endoscopic remission in UC. However, data on its possible antifibrotic effects are lacking. To date, the exact mechanisms of action of 5-ASA are not entirely known. 5-ASA has been shown to inhibit the synthesis of pro-inflammatory prostaglandins and leukotrienes and of several cytokines and to increase the expression of anti-inflammatory molecules such as peroxisome proliferator-activated receptor-γ (PPAR-γ) [5]. None of the tested 5-ASA doses were able to counteract the fibrotic effects induced by TGF-β1 on both CCD18-Co and Caco-2 IEC cells. This suggests that 5-ASA fails to be effective in modulating markers associated with intestinal fibrosis in our in vitro models, and this can be proof of support of the guidelines from the European Crohn’s and Colitis Organisation (ECCO), which does not suggest the use of 5-ASA for the induction and maintenance of remission in Crohn’s disease [23]. 

Immunosuppressive agents have been used consistently for many years in the treatment of several chronic inflammatory diseases, including IBD, but their use did not lead to a reduction in the risk of the development of intestinal fibrostenosis and the consequent inevitable intestinal resections. In addition to this, thiopurine has been associated with an increased risk of lymphoma [24]. In the two in vitro cellular models used in this study, both azathioprine and methotrexate were unable to counteract the fibrotic effects induced by TGF-β1, corroborating the recommendation of the ECCO guidelines that suggest alternative therapies instead of azathioprine and methotrexate [23]. 

Corticosteroids such as prednisone, methylprednisolone, and budesonide are effective in controlling intestinal inflammation and are currently used in the induction of the clinical remission of patients with moderate to severe CD and UC [25], but not in maintaining remission. Although they did not show antifibrotic effects in several fibrotic diseases, such as idiopathic pulmonary fibrosis and systemic sclerosis, in our in vitro models, prednisone and methylprednisolone were able to counteract the increase of collagen I and α-SMA expression in TGF-β1-treated CCD-18Co cells at both doses tested, while budesonide significantly counteracted these effects induced by TGF-β1 only at the highest dose. Notably, the remodeling of the cytoskeleton was also reverted by these steroids. Consistently, prednisone, methylprednisolone, and budesonide were able to antagonize Smad2/3 phosphorylation in CCD-18Co cells. Of the steroids tested, just methylprednisolone and budesonide counteracted the EMT process induced by TGF-β1 in Caco-2 IEC cells, as observed by Western blot and immunofluorescence staining analyses. Despite the favorable response of steroids in both in vitro cellular models, it must be underlined that the long-term use of steroids in patients with IBD is contraindicated by their numerous and relevant adverse events [26,27]. 

Steroid-dependent or refractory IBD patients can benefit from biological drugs. Biological therapies currently used in the treatment of IBD include three main classes of monoclonal antibodies: anti-TNF-α, anti-integrins (α4β7 integrin-receptor antagonist), and anti-interleukin (IL) 12/23. Of these drugs, the anti-integrin and the anti-IL12/23 were excluded from the study due to the absence of the specific targets in the used cell lines [28,29,30,31]. Anti-TNF-α agents were the first biologics approved for the treatment of IBD, including infliximab, adalimumab, golimumab, and certolizumab pegol [28]. Several data have reported the effectiveness of anti-TNF-α agents: two-thirds of CD patients with stenosis had avoided abdominal surgery [32]. Bouhnik et al. [30,33] evaluated the efficacy of adalimumab in patients with stenotic CD, showing its safety and efficacy in maintaining surgery-free remission in half of the included patients over a 4-year follow-up. A cohort study of about ten years of follow-up including patients who had received early treatment with anti-TNF-α agents showed a reduced risk of developing bowel stenosis [34]. On the other hand, Valvano et al. reported that biological therapy had only a slight impact on the eventual occurrence of surgery in CD patients over a long observation period, thus appearing only to delay the first intestinal resection [35]. Despite increased post-operative use of anti-TNFα agents in CD patients in the last two decades, the impact of this strategy on the risk of long-term re-operation rate has been modest [36]. Unfortunately, all biological drugs used in IBD, including monoclonal anti-TNF-α, present a primary non-response in approximately one third of cases and a secondary loss of response in another third of cases (10–20% per year). In the present study, we tested the potential antifibrotic effect of these biologics, evaluating their ability to interfere with the fibrotic cellular phenotype. The anti-TNF agents were included after verifying the TNF-α expression in used in vitro models. As expected, both cell lines (CCD-18Co, Caco-2 IEC) showed basal levels of TNF-α released in the supernatants. As TNF-α can enhance fibrosis through the induction of Metallopeptidase Inhibitor 1 (TIMP-1) expression and the reduction in Matrix Metalloproteinase-2 (MMP-2) activity and collagen degradation [37], anti-TNF-α drugs could have an effective anti-fibrotic action. However, the direct role of these agents as well as the pathways involved in preventing and treating the fibrosis are not fully understood, considering the multiple functions of TNF-α and the role of other cytokines in the fibrotic process, especially of TGF-β1. A recent study highlights the synergistic effects of TNF-α and TGF-β1, demonstrating that these two cytokines were able to induce fibrosis in patient-derived organoids by increasing the expression of EMT-related genes [38]. In our in vitro models, adalimumab lowered the expression of collagen I, α-SMA, and pSmad2/3 in TGF-β1-treated CCD-18Co cells compared to control cells. At the same time, adalimumab was able to revert the TGF-β1-induced EMT process in the Caco-2 IEC cells, upregulating occludin expression and reporting α-SMA levels similar to those of control cells. This result supports the evidence that TNF-α may be a potential target for the treatment of intestinal fibrosis. Surprisingly, and without any plausible explanation, infliximab had no significant effects in both cell systems. The doses of the two biologics used were practically equivalent. The difference between infliximab and adalimumab is that the former is a chimeric monoclonal antibody against TNF-α, while the latter is a human anti-TNF monoclonal antibody. It is not possible to establish whether this structural difference of the two biologics could have had a different influence on the cellular models used in our study and therefore on the different results obtained. However, further investigations are needed to clarify the role of the target cytokine at each stage of the disease. It should also be considered that the widening use of biologics in several autoimmune diseases has been associated with occurrence of a new class of adverse events, called paradoxical immunological reactions, that affect several organs, including the skin, liver, lungs, kidneys, peripheral and central nervous system, vascular system, and bowel [39]. However, from a clinical point of view in IBD, the main limits of anti-TNF are represented by both primary non-response and secondary loss of response.

**Figure 8:**
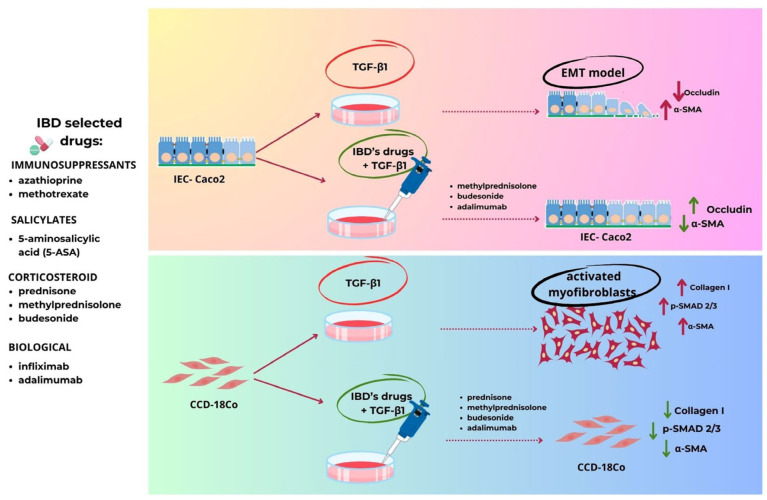
Graphical representation of the main results obtained on the two cell models used in this work.

Taken together, the results of this in vitro study on intestinal cells suggest that steroids such as prednisone, methylprednisolone, and budesonide and the biological adalimumab can exert antifibrotic effects (Figure 8). However, only limited experimental and clinical data have shown the potential antifibrotic effects of the drugs commonly used in the treatment of IBD and only in slowing down the progression of intestinal disease related to the inflammation. Furthermore, the molecular pathways of IBD-associated intestinal fibrosis on which these drugs may act are still unknown. The use of in vitro models such as intestinal cell lines, cell co-cultures, patient-derived organoids, and biopsies, even if they do not mimic the complexity of the in vivo gut, could be useful experimental models to find the key molecular pathways that lead to the switch between inflammation and fibrosis. We are aware that our results derived from in vitro experiments do not imply absolute similarity to what occurs in patients with IBD, however, this work represents the first step of future experiments aimed at deepening these data with in vivo studies using IBD animal models mimicking human intestinal fibrogenesis. Moreover, this study highlights the importance of changing the paradigm in the treatment of IBD, since drugs with increasingly greater anti-inflammatory action are not sufficient to prevent and treat intestinal fibrosis, which frequently complicates this disease. Instead, it is important to test drugs capable of modulating the main cellular and molecular mechanisms of intestinal fibrosis, such as the differentiation of fibroblasts to myofibroblasts and EMT. Therefore, the availability of effective and safe antifibrotic drugs remains an unavoidable clinical challenge and an urgent need for the treatment of IBD-associated intestinal fibrosis. 

## 4. Materials and Methods

### 4.1. Cell Lines and Culture Conditions

CCD-18Co cells, a normal human intestinal fibroblast cell line (ATCC CRL-1459) from American Type Culture Collection (Georgetown, DC, USA), were cultured in Eagle’s Minimum Essential Medium (EMEM) (ATCC) supplemented with 10% of FBS, 100 μg/mL streptomycin, and 100 U/mL penicillin (Euro Clone, West York, UK). After reaching 90% confluence, the cells were detached by trypsin solution (Euro Clone) and seeded into sterile tissue culture 6- or 96-well plates (Becton, San Jose, CA, USA) at 5000 cells/cm^2^ and treated as reported below. The Caco-2 cell line was purchased from Sigma-Aldrich and then cultured in Dulbecco’s modified Eagle’s medium (DMEM) supplemented with 10% fetal bovine serum (FBS), 100 U/mL penicillin, 1 mM sodium pyruvate, 2 mM L-glutamine, 1% non-essential amino acids, and 100 μg/mL streptomycin (Euro Clone). After reaching 80% confluence, cells were detached by trypsin solution (Euro Clone) and seeded into sterile tissue culture 12- or 96-well plates (Becton) at 60,000 cells/cm^2^. As previously described [40,41,42] to obtain intestinal epithelial differentiated cells, Caco-2 cells were maintained in culture for fourteen days post-confluence and then treated as reported below. The cell cultures were maintained in a humidified atmosphere of 95% air and 5% CO_2_ at 37 °C.

### 4.2. Cell Models and Treatments

CCD-18Co cells plated at 5000 cells/cm^2^ were serum-deprived for 24 h and then treated with 10 ng/mL of human transforming growth factor (hTGF-β1; Cell signaling Technology, Danvers, MA, USA) for 48 h to induce the fibrotic phenotype in the presence or absence of mesalamine (Sigma-Aldrich, St. Louis, MO, USA), azathioprine (Azafor, Sofar, MI, Italy), prednisone (Deltacortene, Bruno Pharmaceuticals, RM, Italy), methylprednisolone (Urbason, Sanofi, MI, Italy), budesonide (Intesticort, Sofar, MI, Italy), methotrexate (Reumaflex, Alfasigma, BO, Italy), infliximab (Flixabi, Biogen, Cambridge, MA, USA), and adalimumab (Hyrimoz, Sandoz, Priceton, NJ, USA). All drugs were previously dissolved and diluted to reach the tested concentrations in the culture medium. To establish an epithelial-to-mesenchymal transition in vitro model, Caco-2 IEC cells were stimulated with 20 ng/mL TGF-β1 in the serum-free medium up to 96 h in the presence or absence of the above drugs. Then, the cells were harvested and centrifuged at 400× *g* for 10 min. The pellets were incubated with a 0.04% Trypan blue (Euro Clone) solution for 5 min to analyze cell number and viability. Non-treated cells served as controls. The cells were counted by microscopy (Eclipse 50i, Nikon Corporation, Tokyo, Japan).

### 4.3. Cell Viability Assay

Cell viability was measured using the Cell Counting Kit-8 (CCK-8) (Sigma-Aldrich) according to the manufacturer’s instructions. The 96-well-plated CCD-18Co cells were treated for 48 h with different concentrations of the selected drugs: mesalamine (0.1–5 mM), azathioprine (5–20 µM), prednisone (10–100 µM), methylprednisolone (50–200 µM), budesonide (0.1–5 µM), methotrexate (0.1–1 µM), infliximab (10–50 µg/mL), and adalimumab (20–100 µg/mL). Similarly, the 96-well-plated Caco-2 cells were incubated for up to 96 h with mesalamine (5–50 mM), azathioprine (1–20 µM), prednisone (10–200 µM), methylprednisolone (10–200 µM), budesonide (7–70 µM), methotrexate (1–50 µM), infliximab (25–200 µg/mL), and adalimumab (8–100 µg/mL). After incubation, 10 µL of CCK-8 assay reagent was added to each well. The optical density (OD) values were measured using a spectrophotometer (BioRad, Hercules, CA, USA) at 450 nm.

### 4.4. Western Blot Analysis

For Western blot analyses, cell pellets were collected, washed in PBS, and then lysed in RIPA buffer (Merck KGaA, Darmstadt, Germany) containing a protease inhibitor mixture (5 μg/mL carboxypeptidase inhibitor, 5 μg/mL trypsin inhibitor, 1 mM PMSF, 10 μg/mL leupeptin, 10 μg/mL aprotinin, and 10 μg/mL pepstatin) (Sigma Aldrich). The protein content of samples was then measured with DC Protein Assay (BioRad) using BSA as a standard. Subsequently, 25 μg of proteins were mixed with the sample buffer, boiled for 5 min at 100 °C, and then separated by 10% SDS-polyacrylamide gel electrophoresis. The transfer of proteins was performed onto a 0.45 μm nitrocellulose membrane sheet (BioRad), for 1 h at 4 C at 70 V, using a Mini Trans-Blot Cell apparatus (BioRad). Blocking of membranes was done by soaking the membrane in 5% non-fat dry milk for 1 h at room temperature. Overnight incubation was done at 4 °C with the following antibodies: rabbit polyclonal antibody anti-COL1A1 (Boster Biological Technology, Pleasanton, CA, USA) 1:1000, rabbit monoclonal antibody anti-human phospho-SMAD2(Ser465/467)/SMAD3(Ser423/425) (Cell Signaling Technology) 1:1000, rabbit polyclonal antibody anti-OCCLUDIN (OriGene, Rockville, MA, USA) 1:1000, mouse monoclonal antibody anti-α-actin smooth muscle (ACTA2, α-SMA) (OriGene) 1:1000, and mouse monoclonal antibody anti-GAPDH (OriGene) 1:1000. Horseradish peroxidase (HRP)-conjugated goat anti-rabbit IgG secondary antibody at 1:2000 was used for anti-COL1A1, anti-human phospho-SMAD2/3, and anti-occludin antibodies and horseradish peroxidase (HRP)-conjugated rabbit anti-mouse IgG secondary antibody at 1:2000 for anti-α-SMA and anti-GAPDH antibodies (Millipore EMD, Darmstadt, Germany). Immuno-reactive protein bands were visualized by enhanced chemiluminescence (ECL, Amersham Pharmacia Biotech, Amersham, UK) according to the manufacturer’s instructions. Band densities were determined using the ALLIANCE (UVITEC, Cambridge, UK) chemiluminescence documentation system and normalized to the relative GAPDH bands. Values were given as relative units.

### 4.5. Immunofluorescence Staining

CCD-18Co and IEC Caco-2 cells, grown on coverslips in a 12-well plate (seeded at 5000 cells/cm^2^ and 60,000 cells/cm^2^, respectively), were treated as previously described. The coverslips were then washed with PBS, fixed with 4% formaldehyde for 20 min, and permeabilized in 0.1% triton X-100 (Sigma-Aldrich) for 5 min. Following fixation, coverslips were blocked with 3% BSA (Sigma-Aldrich) for 20 min at room temperature. Subsequently, cells were incubated overnight at 4 °C with rabbit polyclonal antibody anti-COL1A1 (Boster Biological Technology) 1:250, mouse monoclonal antibody anti-α-actin smooth muscle (ACTA, α-SMA) (OriGene ) 1:250, and rabbit polyclonal antibody anti-OCCLUDIN (OriGene) 1:50. Therefore, staining was performed using FITC conjugated goat anti-mouse polyclonal IgG secondary antibody (Bethyl Laboratories, Inc, Montgomery, TX, USA) 1:1000 or FITC conjugated goat anti-rabbit polyclonal IgG secondary antibody (Millipore) 1:1000 for 1 h at room temperature, washed, and then incubated with TRITC labeled phalloidin (Sigma-Aldrich) for 45 min at room temperature. The coverslips were mounted with VECTASHIELD^®^Antifade Mounting Medium with DAPI (Vector Laboratories, Inc., Burlingame, CA, USA) and then examined at 40× magnification with a fluorescent microscope (Eclipse 50i, Nikon, Tokyo, Japan).

### 4.6. TNF-α ELISA Assay

The levels of released TNF-α were detected in the cell supernatants using an enzyme-linked immunosorbent assay (ELISA) kit (Immundiagnostick, Bensheim, AG, Germany). Briefly, the CCD-18Co cells were plated at 5000 cells/cm^2^, grown overnight, and then serum-deprived for 48 h, while the Caco-2 IEC cells were plated at 60,000 cells/cm^2^, and fourteen days post-confluence, they were cultivated in the serum-free medium up to 96 h. After the media were collected and cleared of cellular debris/dead cells by centrifugation at 600× *g* for 10 min, the TNF-α concentration was quantified, and the data obtained were normalized for cell number and expressed as pg/10^5^ cells.

### 4.7. Statistical Analysis

All data were evaluated by GraphPad Prism (version 6.01 GraphPad Software, San Diego, CA, USA). One-way ANOVA or two-way ANOVA, followed by Dunnett’s post hoc test, was used to compare the mean values among groups. Data were expressed as mean ± SD or mean ± SEM. *p* < 0.05 was considered statistically significant.

## Data Availability

All data generated or analyzed during this study are included in this article; further inquiries can be directed to the corresponding author.
